# Versatility of nodal affiliation to communities

**DOI:** 10.1038/s41598-017-03394-5

**Published:** 2017-06-27

**Authors:** Maxwell Shinn, Rafael Romero-Garcia, Jakob Seidlitz, František Váša, Petra E. Vértes, Edward Bullmore

**Affiliations:** 1 Department of Psychiatry, Behavioural and Clinical Neuroscience Institute, University of Cambridge, Cambridge, CB2 0SZ United Kingdom; 2 Developmental Neurogenomics Unit, National Institute of Mental Health, Bethesda, MD 20892 USA; 3 GlaxoSmithKline Clinical Unit Cambridgeshire & Peterborough NHS Foundation Trust, Cambridge, Addenbrookes Hospital, Cambridge, CB2 0QQ United Kingdom; 4 GlaxoSmithKline R&D, Immunology & Inflammation Therapeutic Area Unit, Stevenage, SG1 2NY United Kingdom

## Abstract

Graph theoretical analysis of the community structure of networks attempts to identify the communities (or modules) to which each node affiliates. However, this is in most cases an ill-posed problem, as the affiliation of a node to a single community is often ambiguous. Previous solutions have attempted to identify all of the communities to which each node affiliates. Instead of taking this approach, we introduce *versatility*, *V*, as a novel metric of nodal affiliation: *V* ≈ 0 means that a node is consistently assigned to a specific community; *V* >> 0 means it is inconsistently assigned to different communities. Versatility works in conjunction with existing community detection algorithms, and it satisfies many theoretically desirable properties in idealised networks designed to maximise ambiguity of modular decomposition. The local minima of global mean versatility identified the resolution parameters of a hierarchical community detection algorithm that least ambiguously decomposed the community structure of a social (karate club) network and the mouse brain connectome. Our results suggest that nodal versatility is useful in quantifying the inherent ambiguity of modular decomposition.

## Introduction

The community structure of a network divides the network into groups, or communities, which share topological similarity. These communities are most commonly defined to be non-overlapping groups which maximise the strength of edges within the community and minimise the strength of edges which leave the community, where each node is a member of one and only one community.

Sometimes, the community structure of a network is evident even to an untrained observer. It is very clear which nodes belong to which community, and which nodes and edges are involved in linking communities together. In other words, the overall community structure is unambiguous for nearly all of the nodes in the network.

However, in most networks, the modular decomposition of community structure is an ill-posed problem, as not all nodes can be assigned unambiguously to a single community. Techniques previously developed to deal with this situation include algorithms that allow overlapping communities^1^ and algorithms that work not with communities themselves, but rather with probability distributions of communities via multi-layer networks^2^. These approaches, while attractive in theory, can be challenging to operationalise and do not facilitate an intuition about the underlying structure of the network. Various forms of consensus clustering^3, 4^ have been developed to optimise non-overlapping modular decomposition “on average” over an ensemble of datasets or runs of a non-deterministic community detection algorithm. However, it remains debatable whether these communities represent the “true” communities of the network, or just the best possible consensus solution given the algorithm and the available data.

Our approach to the issue of community ambiguity is predicated on the observation that, although the community structure of a network may not be certainly known, there will generally be variability between nodes in terms of the certainty with which they can be individually affiliated with a specific community. Here, we seek to formalise this intuition by developing a new metric called *versatility* which can be used to quantify the certainty with which each node is assigned to the same community of a network. Versatility may be computed with respect to any stochastic community detection algorithm, and therefore provides a measure of how the algorithm interacts with the network on a nodal level.

In what follows, we first define an estimator of nodal versatility of community affiliation and demonstrate its desirable properties by analysis of idealised networks designed for maximal community ambiguity. To build intuitive understanding of what versatility is measuring, we explored its performance in two real-life networks: the karate club graph, a social network; and the mouse brain connectome, a brain network derived from anatomical tract-tracing experiments. In both of these cases, we show how versatility can be used to identify the resolution parameters of the Louvain hierarchical community detection algorithm^5^ that provide the least ambiguous modular decomposition of the network as a whole. Additionally, we use versatility to characterise the topological roles of each individual node.

## Methods

We propose a measure of nodal versatility of community affiliation that can be estimated for any graph (weighted or unweighted, directed or undirected), and for any non-deterministic or stochastic algorithm which decomposes the community structure of such graphs.

### Definition of versatility

The most natural way to accurately capture the intuition of variability in community classification with respect to an arbitrary algorithm applied to a single graph is to run a stochastic community detection algorithm many times. Because the partitions will be different, we use as our fundamental quantity the probability *p*_*i*,*j*_ that any two nodes *i* and *j* will be classified in the same community. This is equivalent to the element in the *i*’th row and the *j*’th column of the association matrix from consensus clustering^3^. If two nodes are always in the same community, *p*_*i*,*j*_ will be equal to 1, and if they are never in the same community, it will be 0. Likewise, if they are in the same community 50% of the time, *p*_*i*,*j*_ will be equal to 0.5. Versatility should be highest for a node *j* when *p*_*i*,*j*_ = 0.5 for all *i* ≠ *j*. To formalise this idea, we transform the *p*_*i*,*j*_ values with the sine function, and then sum the transformed *p*_*i*,*j*_ values for each node *j*. Finally, we normalise by the average number of nodes in the community containing node *j*. So, for node *j*, we sum sin(*πp*_*i*,*j*_) for all nodes *i* and normalise by dividing by the mean size of the communities containing *j* (weighted by membership probability).

More formally, the versatility of a node *j* is defined as

1 $$V(j)=\frac{\sum _{i}\sin (\pi {\mathbb{E}}(a(i,j)))}{\sum _{i}{\mathbb{E}}(a(i,j))},$$

where ${\mathbb{E}}$ is expected value, and

$$a(i,j)=\{\begin{array}{cc}1 & i\,{\rm{a}}{\rm{n}}{\rm{d}}\,j\,{\rm{a}}{\rm{r}}{\rm{e}}\,{\rm{i}}{\rm{n}}\,{\rm{t}}{\rm{h}}{\rm{e}}\,{\rm{s}}{\rm{a}}{\rm{m}}{\rm{e}}\,{\rm{c}}{\rm{o}}{\rm{m}}{\rm{m}}{\rm{u}}{\rm{n}}{\rm{i}}{\rm{t}}{\rm{y}}\\ 0, & {\rm{e}}{\rm{l}}{\rm{s}}{\rm{e}}\end{array}.$$

**Figure 1:**
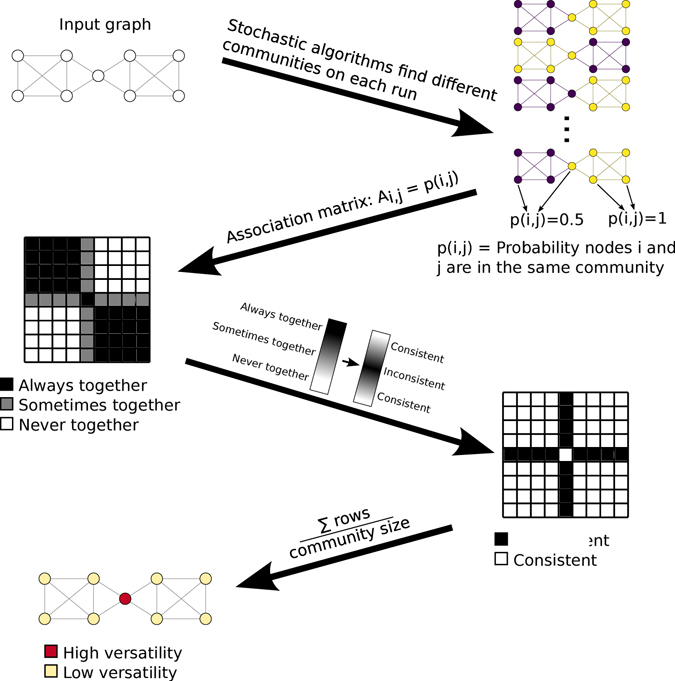
A schematic overview of versatility. A stochastic community detection algorithm is run many times, generating a collection of graph partitions. Each pair of nodes corresponds to a cell in the association matrix, which describes the sample probability that any two nodes will be grouped in the same community. A transformation function is applied to this matrix, so that pairs of nodes that are consistently grouped either in the same community or in a different community are given low values, and pairs which are only sometimes in the same community are given high values. The sum is taken for each node’s possible pairs, and normalised by the mean size of the communities weighted by the node’s community membership. The resulting normalised sum is the node’s versatility.

Because ${\mathbb{E}}(a(i,j))={p}_{i,j}$, we estimate the expected value of *a*(*i*, *j*) by running any given community detection algorithm many times. See Fig. 1 for a graphical summary of this process.

This formula for versatility can be computed for any stochastic community detection algorithm. If versatility is being used to understand a community decomposition, the same algorithm and parameters should be used for each iteration as for the original decomposition. For algorithms based on Newman’s quality function *Q* ^6^ such as the Louvain algorithm^5^, or for any other algorithm with a resolution parameter, computing versatility across many different resolution parameters can offer insight into which values of this parameter minimise the ambiguity of the modular decomposition.

For the Louvain algorithm, we have determined that approximately 1000 runs at a particular resolution parameter gives reliable results in our networks, based on numerical simulations tracking the variance in versatility across multiple runs in the same network (Supplementary Fig. S1). This number may be higher or lower in different networks or with other community detection algorithms. Though versatility can be computed for any community detection algorithm, we use the Louvain algorithm in what follows due to its popularity in the literature. Code to calculate versatility for any arbitrary community detection algorithm is available in Python and Matlab/Octave from https://github.com/mwshinn/versatility. This includes code to generate a plot which can be used to find an optimal resolution parameter.

### Technical evaluation of versatility estimators

**Figure 2:**
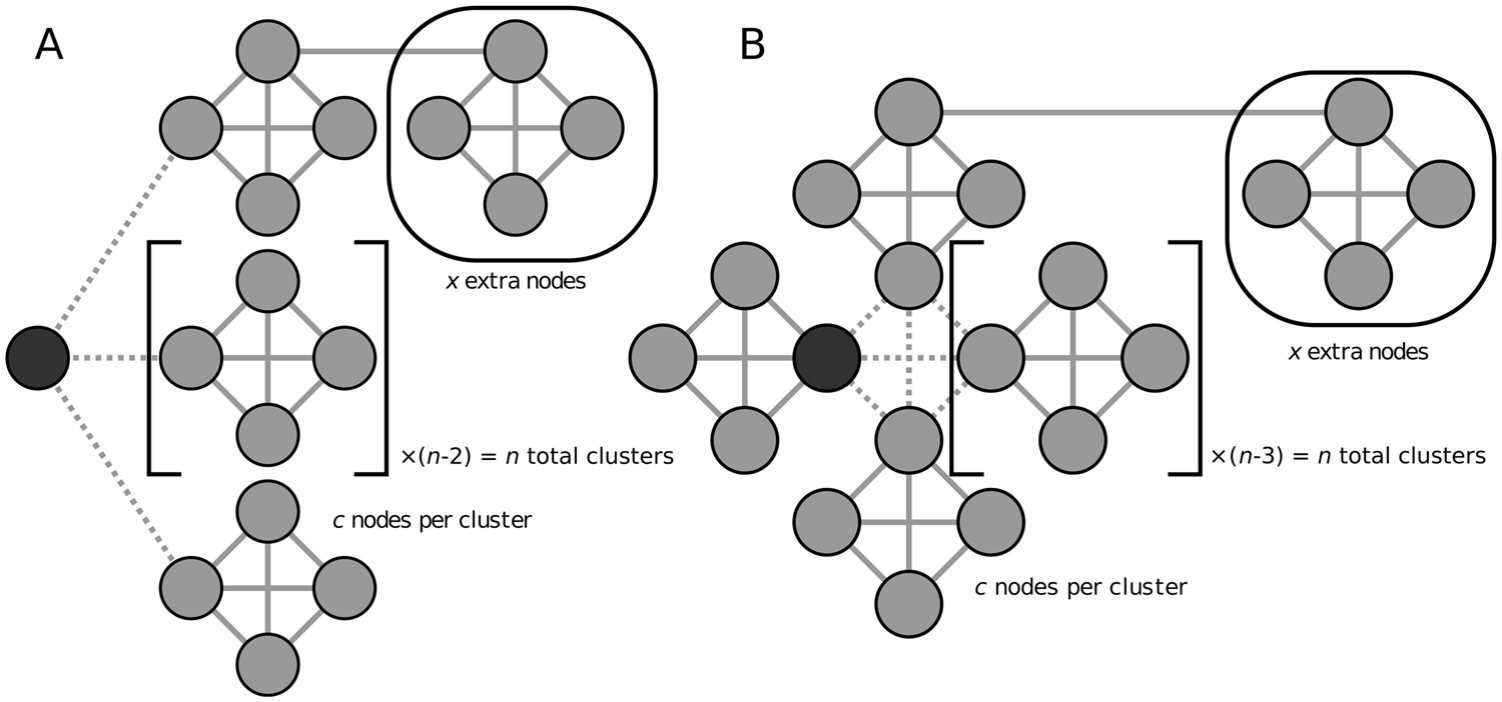
Test networks for evaluation of candidate versatility metrics. (**A**) The n-split case. An indicator node (coloured darker) can be affiliated with exactly one of the *n* clusters with probability 1/*n*. (**B**) The n-clusters case. There are *n* clusters each with a probability *p* of connecting to any other given cluster. The indicator node is a member of one of these clusters which is weakly connected to other clusters. In both cases clusters are of size *c*, and these cases are assumed to be a part of a larger network, with *x* other nodes.

In theory, there are several ways in which versatility of nodal affiliation to a modular community structure could be defined. To choose between the many versatility estimators that are potentially available, we first list the desirable properties of a theoretically optimal estimator and then evaluate a number of candidate estimators against these criteria, using two test networks to assess the performance of each estimator empirically, as shown in Fig. 2.

#### Model networks

The first model is called the “n-split network” and represents the case where a single *indicator node* has one connection to each of a number of identical tightly-interconnected clusters. It has three parameters: *n*, the number of clusters to which the indicator node is connected; *c*, the number of nodes in each cluster; and *x*, additional nodes in the network that change the total network size but are assumed to not affect the community detection. Thus, the total size of the network is *n* × *c* + *x* + 1. We assume that the indicator node can be assigned to only one community by each run of a stochastic community detection algorithm, with the probability of affiliation to each model on each run being equal to 1/*n*.

The second model is called the “n-clusters network” and represents the case where there are several identical clusters in the network which are each tightly interconnected within themselves, but only loosely (and symmetrically) connected to each other. This model has four parameters which echo those of the n-split case: *n*, the number of clusters we are to consider in the network; *c*, the number of nodes in each cluster; *x*, the number of additional nodes in the network that change the total network size but are assumed to not affect the community detection; and *p*, the probability that, in any given run of an ideal community detection algorithm, any two clusters will be grouped in the same community. The indicator node is taken to be an arbitrary node in an arbitrary cluster, which are assumed to have identical properties due to symmetry. The indicator node is connected to a cluster with a total of *c* nodes, and this *indicator cluster* is connected with probability *p* to *n* − 1 other clusters each of size *c*. In any given run of an ideal community detection algorithm, the indicator cluster can be assigned its own unique community or it can be affiliated to a larger community also comprising one or more of the other clusters, each with probability *p*. Thus the indicator cluster will be affiliated to the same community as at least one other cluster with probability 1 − (1 − *p*)^*n*^, and it will be affiliated to the same community as all of the other clusters with probability *p* ^*n*^.

It is important to note that these networks are strictly theoretical. It is unknown whether any given community detection algorithm would actually exhibit the partitions described above. For example, in practice, the Louvain algorithm behaves deterministically in the n-split case, preferring always to affiliate the indicator node with the same community based on the algorithmic bias caused by the order of the adjacency matrix. However, the behaviours described above are what we would expect if the model networks were decomposed into a community structure by a stochastic community detection algorithm which exhibits the idealised behaviours described in conjunction with each network.

#### Desirable properties of versatility metrics

In this context, we can list the key desirable properties of a versatility metric as follows:

**Lower bound:** Versatility should be bounded below by 0.

**Upper bound as network size**→∞**:** A universal upper bound on versatility should exist regardless of network size.

**Degree invariance:** The degree of a node, or the number of connections it makes, should not directly affect its versatility.

**n-split zero:** If the number of clusters *n* = 1 in the n-split network, the versatility of the indicator node is 0.

**n-cluster zero:** If the probability of being grouped with another cluster *p* = 0 or 1 in the n-cluster network, the versatility of the indicator node is 0.

**Network size invariance:** Versatility is unaffected by the total number of nodes in the network, represented by *x* in both the n-split and n-cluster networks.

**n-split cluster size monotonicity:** Versatility is an increasing function of the number of nodes per cluster *c* in the n-split network.

**n-cluster cluster size invariance:** Versatility is unaffected by the number of nodes per cluster *c* in the n-cluster network.

**Cluster number monotonicity:** Versatility is an increasing function of the number of clusters *n* in both networks.

**Splitting over breaking:** All else being equal, a node probabilistically connected to two communities should have higher versatility than a node in a cluster that is probabilistically contained within the same community as other clusters. So for otherwise equal networks, the n-split case should result in higher versatility than the n-cluster case.

#### Candidate versatility metrics

We evaluated six candidate versatility metrics that were expected to be reasonably well-behaved. They followed the general form

2 $$V(j)=\frac{1}{g(j)}\sum _{i}\,f({\mathbb{E}}(a(i,j))),$$

where *a*(*i*, *j*) is a stochastic function that measures whether nodes *i* and *j* are in the same community; *g* : {1, 2, …, *N*} → (0, ∞) is a normalisation function for a network with *N* nodes; ${\mathbb{E}}$ is the expected value; and *f* : [0, 1] → [0, 1] is any continuous, concave function that has the values *f*(0) = 0, *f*(1) = 0, and *f*(0.5) = 1, and is symmetric around 0.5, i.e. *f*(*x*) = *f*(1 − *x*).

Three functions were chosen for *f* and two for *g*, and each was denoted by a capital letter; the six possible combinations of *f* and *g*, each denoted by a two-letter code, represented the six versatility metrics tested. For *f*, we evaluated the sine function,

$$f(x)\,=\,\sin (\pi x)\equiv {\rm{S}},$$

the entropy function,

$$f(x)=-x{{\rm{l}}{\rm{o}}{\rm{g}}}_{2}(x)-(1-x){{\rm{l}}{\rm{o}}{\rm{g}}}_{2}(1-x)\equiv {\rm{E}},$$

and the triangle wave function,

$$f(x)=\{\begin{array}{cc}x, & 0\le x\le 0.5\\ 1-x, & 0.5\le x\le 1\end{array}\equiv {\rm{T}}.$$

For *g*, we tried normalising by the number of nodes in the network

$$g(j)=\sum _{i}1\equiv {\rm{U}},$$

and also by the mean community size

$$g(j)=\sum _{i}{\mathbb{E}}(a(i,j))\equiv {\rm{C}}.$$

While we could not examine the full space of potential functions *f* and *g*, we considered those that seemed most natural given the constraints. We did not consider metrics that were normalised by the degree of the node, because this breaks the intuition that versatility should depend only on the community classification. We also did not explore metrics which depend only on the probability of the indicator node being in the same community as its nearest neighbours, rather than all nodes, as these by definition do not satisfy the degree invariance property. Such metrics may be more suitable as a community-agnostic version of participation coefficient, rather than assessing the degree to which a node affiliates with a community.

#### Participation coefficient

Versatility was contrasted with participation coefficient^7^, because in informal terms, both describe the coupling of a node with its community. Participation coefficient tries to measure the intuitive property of whether nodes could facilitate communication across separate groups of nodes by having a high inter-community degree. It is defined as

3 $${\rm{\text{PC}}}(i)=1-\sum _{s=1}^{K}\frac{{k}_{i,s}}{{k}_{i}},$$

where *K* is the number of communities, *k*_*i*_ is the degree of node *i*, and *k*_*i*,*s*_ is the intra-community degree of node *i*.

#### Performance of candidate metrics

Each of the 6 candidate versatility metrics was benchmarked by its performance in analysis of the two model networks as shown in Table 1, where they are compared to participation coefficient (PC).

**Table 1:** A list of desirable properties satisfied by each of the algorithms.

Metric	SU	EU	TU	SC	EC	TC	PC
Lower bound	0	0	0	0	0	0	0
Upper bound as network size →∞	1	1	1	π	∞	2	1
Degree invariance	✓	✓	✓	✓	✓	✓	
n-split zero	✓	✓	✓	✓	✓	✓	✓
Network size invariance				✓	✓	✓	✓
n-split cluster size monitonicity	✓	✓	✓	✓	✓	✓	*
n-cluster cluster size invariance	✓	✓	✓	✓	✓	✓	
n-split cluster number monotonicity				✓	✓	✓	†
n-cluster cluster number monotonicity	✓	✓	✓	✓	✓	✓	†
Splitting over breaking	✓	✓	✓	✓	✓	✓	

✓ Indicates satisfying the property. Numeric values are listed where relevant. * Denotes trivially satisfying the monotonicity properties by being constant functions. † Denotes that participation coefficient trivially satisfies certain properties due to the lack of degree invariance. Upper bounds were found analytically (see Supplementary Information).

Normalisation by the size of the network (U) was not effective. None of the metrics SU, EU and TU satisfied the desirable property of network size invariance. Thus these metrics were not considered further. The three metrics normalised by community size (SC, EC, TC) were much more evenly matched. Because TC only trivially satisfies n-split cluster monotonicity, we consider only SC and EC. In practice, SC and EC give nearly identical results in all networks for which they have been computed. SC and EC are very similar and would both make good measures of versatility; however, we select SC for two reasons. First, and most importantly, it has an upper bound. Second, it avoids the potential for confusion created by using the formula for Shannon’s entropy, when in fact no information-theoretic quantity is involved^8^. Thus, SC is equivalent to Eq. 1.

While the versatility of any given node depends only on community affiliation and does not depend on the size of the network, one intriguing aspect of SC is that the maximum possible versatility a node is capable of achieving in a network does depend on the network size. This is due to the fact that increasing the size of the network increases the number of nodes that could potentially be in the same community as the indicator node. In finite networks with *N* nodes, we can construct a network which maximises versatility of the indicator node by considering an n-split network with clusters of size *c* = 1, with a total of *n* = *N* − 1 clusters and *x* = 0 extra nodes. Supplementary Fig. S2 shows the maximum versatility as a function of network size. Only for networks with infinitely many nodes can SC reach its maximum of *π* (see Supplementary Information for proof).

We elected not to normalise versatility by *π* because the maximum versatility in any network with a finite number of nodes will be less than *π*, with the limit depending on the number of nodes. Thus, it would be misleading to imply that the maximum versatility is 1 in a finite-sized network. Furthermore, versatility in the un-normalised form has other distinctive landmarks. For example, a versatility of 2.0 means that a node is perfectly split between two equally-sized communities (as the community size approaches infinity).

We also chose not to normalise versatility by the upper bound given the network size (the curve in Supplementary Fig. S2). Doing so would create a dependence on network size, and thus no longer satisfy the desirable properties listed above. An additional problem with normalising is that the maximum versatility given the network size currently must be calculated numerically, and thus the difficulty of implementing versatility would increase. Furthermore, a conceptual problem with normalising by the maximum given the network size is that it cannot possibly be as difficult to classify a node in a 2-node network as it is to classify a node in a 1000-node network; in the former case there are only two possible partitions for the network, whereas in the latter there are approximately 10^1927^ (the 1000th Bell number).

Thus, due to the desirable properties of SC, the definition of versatility is as given in Eq. 1.

## Results

### Versatility can find optimal modular resolution parameters

Initially, Girvan and Newman’s *Q* modularity did not include a resolution parameter^6^; only later was such a parameter *γ* added to search for community structure across a range of community sizes, from a few large communities (low *γ*) to a larger number of smaller communities (high *γ*)^9^. As a result, *Q* values are highly dependent on the resolution parameter, not just in terms of the network topology, but also in terms of the expected magnitude of *Q* for highly modular networks^10^. Thus, directly maximising *Q* across values of *γ* will not provide insight into the structure of the network, making it difficult to use *Q* as an objective function to determine an “optimal” resolution of the hierarchical community structure or value of *γ*.

By contrast, a network’s mean nodal versatility depends only on how consistently the nodes in the network affiliate with a specific community. Thus, we could define an “optimal” value of *γ* as one for which the versatility is lowest, or for which the community structure of the network is least ambiguously defined. The proposed versatility-based procedure provides information about the effectiveness of different resolution parameters allowing the experimenter to make a principled choice.

When the global mean versatility is plotted at each value of *γ* within a reasonable range, it varies as a function of the resolution parameter and there are typically one or more values of *γ* corresponding to local minima in global mean versatility. At the extremes of the curve, the versatility will be zero, as these represent the cases of either a single community encompassing the entire network, or each node in the network being in a separate community. While these parameter ranges are the global minimum of versatility, they are not desirable because they do not take into account the purpose of the community decomposition itself, i.e. to find useful communities.

In order to balance practical considerations with a principled method of using the versatility curve to select optimal *γ*, one of several approaches may be taken. One can choose the value of *γ* which globally minimises the ambiguity in modular classification among those values of *γ* which generate non-trivial community structure. In other words, this finds a community structure which maximises the ability of the algorithm to assign nodes to communities. One can also choose the value of *γ* within a range of resolution parameters that gives a theoretically expected number of communities, or which satisfy another practical requirement. For instance, in brain networks where multiple modalities can be used to define different networks in the same subject^11^, it may be most useful to find equal numbers of communities in each subject to compare across modalities. Additionally, many networks exhibit a region of *γ* values for which versatility is consistently low across many nearby resolution parameters. Even if it does not globally minimise versatility, this scheme prioritises the stability of the mean versatility across small perturbations in resolution parameter.

Recent work^12–14^ has used the number of communities as a proxy, but this method assumes that nodes change community membership only when the number of communities changes; indeed, versatility allows for a more precise selection of resolution parameter (Supplementary Fig. S3). Others have used the z-score of the Rand coefficient^10, 15^, which provides similar resolution parameter suggestions as versatility in some cases (Supplementary Fig. S4). However, it does not provide information on which nodes are driving this change.

The important point is that the global mean versatility curve provides an objective function to guide the otherwise unrestricted and unprincipled choice of resolution parameters often corresponding to different community structures. By understanding which resolution parameters minimise ambiguity, we can make a more informed and precise selection.

### Evaluation on the karate club network

Zachary’s karate club graph^16^ is a non-trivial benchmark and standard test case for community detection algorithms. This network represents friendships in a university karate club before a political conflict caused the club to split into two: members of the club are nodes and friendships between members are edges. Most community detection algorithms are able to find two distinct groups of individuals in the club which correspond to the two political factions in the club after the split. However, there is one individual who has exactly one friendship on one side of the split and one friendship on the other side. Community detection algorithms are forced to assign this individual node to only one of the two communities.

**Figure 3:**
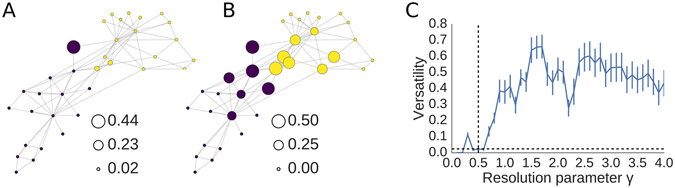
Versatility in the karate club network. (**A**) The versatility in Zachary’s karate club network is compared to (**B**) the participation coefficient in the same network, where the size of the node represents the versatility or the participation coefficient, respectively. Versatility is only high for the nodes in between communities, whereas participation coefficient is also high for the hubs since they tend to have more edges into the other community. The node with very high versatility has exactly one edge in each community. Nodes are coloured according to their community with a resolution parameter of 0.5, which is shown in (**C**) to minimise the mean versatility, i.e. providing the most stable communities. Participation coefficient in (**B**) was also calculated according to this partition structure. We know *a priori* that this club split from one group into two factions, and indeed this range of resolution parameter gives two communities. Error bars represent SEM.

As we see in Fig. 3A, most of the nodes have low versatility, except the individual with one friendship in each of the communities. Nodes that are connected to only one group cleanly sort into their respective faction. Versatility distinguishes itself from participation coefficient shown in Fig. 3B by only holding a high value for the nodes which could be classified into either community by the algorithm. Participation coefficient, by contrast, highlights the degree to which a node is an inter-community hub. We might expect the individuals with high participation coefficients to make effective mediators in this conflict or to be ambassadors between the factions. By contrast, we would expect the individual with high versatility to have a difficult decision on which of these two clubs to join after the split occurred. This exemplifies an important difference between participation coefficient and versatility.

When we look at the curve of versatility across different resolution parameters *γ* in Fig. 3C, we see versatility is near-zero around *γ* = 0.5. This happens to be a parameter which divides the network into two communities, consistent with the prior knowledge that this social network was indeed divided into two communities^17^.

### Mouse connectome

With recent advances in biotechnology, it has become possible to use graph theory based techniques to study the brain^11^. Such work has shown promise in helping us understand concepts ranging from locomotion in model organisms^18^ to diseases as complex as schizophrenia^19^. Since the brain has long been hypothesised to function as a set of semi-independent modules^20^, and the communities of brain networks obtained using graph theory have anatomical and functional significance^21^, it is only natural to talk about brain modules as communities in brain networks^22^.

While there are many ways to find which regions of the mammalian brain are connected, one of the most reliable ways is by injecting a fluorescent viral tracer into a source region of the brain. In the days following injection an anterograde viral tracer travels along the axons of neurons projecting from the source region to anatomically connected target regions. By measuring the strength of the fluorescent tracer in high resolution microscopic images of the injected animal’s brain, it is possible to quantify the weight of anatomical connectivity from a source region to each possible target region. When this experiment is performed in many different mice, with different source regions injected in different experiments, the complete anatomical connectivity matrix or connectome of the mouse brain can be estimated^23^.

A weighted, directed network was constructed from 112 brain regions of the mouse brain connectome derived from over 400 of such tract-tracing experiments conducted by the Allen Institute for Brain Sciences^23^, as previously described^14^.

**Figure 4:**
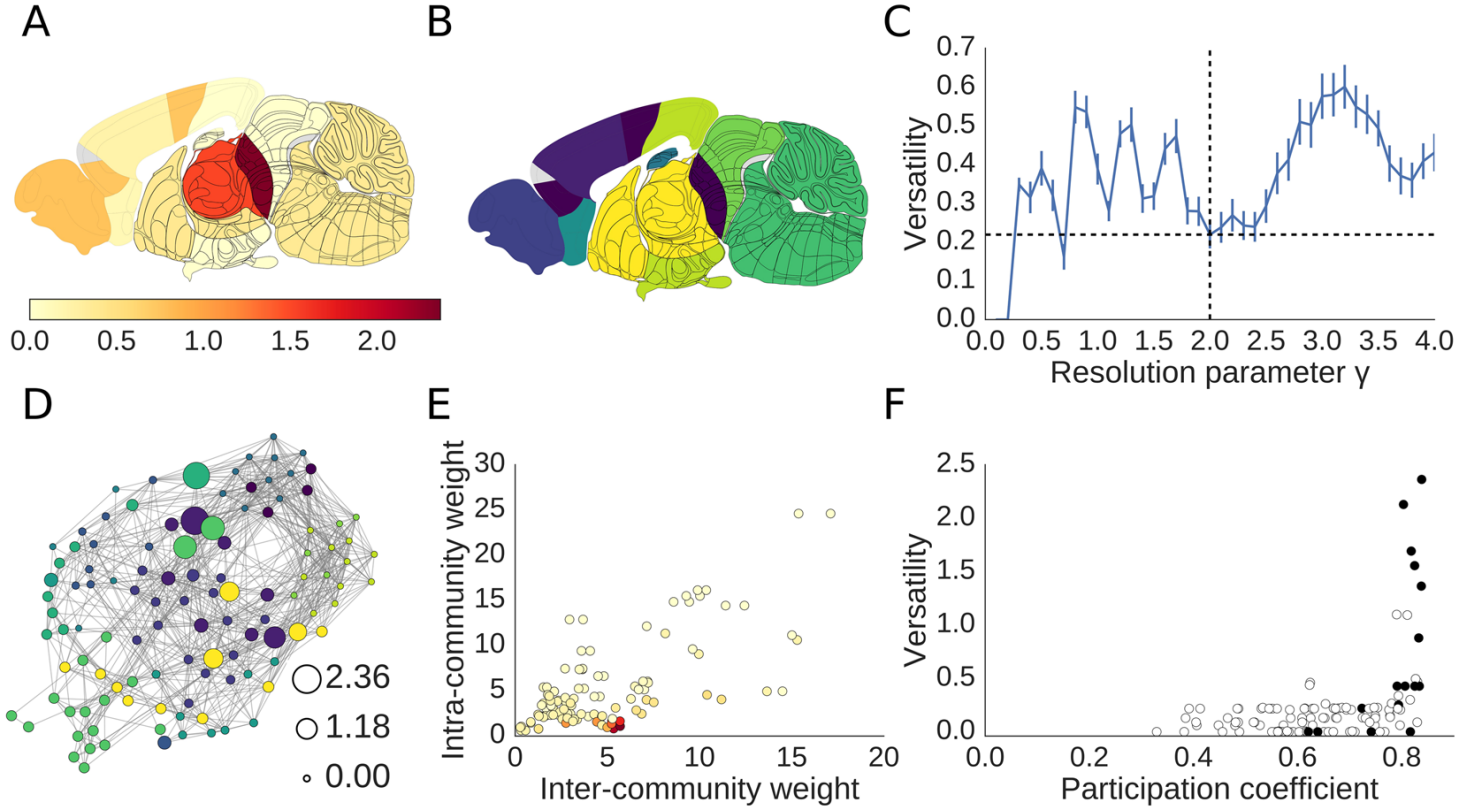
Versatility in the mouse connectome. (**A**) The versatility of each region in the mouse brain, plotted anatomically. (**B**) An example classification of the communities in mouse at an optimal resolution parameter, determined using the curve shown in (**C**). Error bars represent SEM. (**D**) A topological view of the mouse connectome. Nodes are coloured by community using the colour scheme from (**B**). The mean versatility is given by the size of the node. (**E**) Versatility is related to inter-community weight and intra-community weight. Highly versatile nodes have low intra-community weight and high inter-community weight. Versatility, indicated by colour, is the mean versatility across resolution parameters. Colours correspond to the colour bar in (**A**). (**F**) Versatility is plotted against participation coefficient, where participation was computed at the resolution parameter from (**C**). Previous work^14^ identified several nodes which they called “hi-par” nodes (meaning “high participation”), which are coloured black. The mean versatility of the hi-par nodes is significantly (Wilcoxon-Mann-Whitney *p* < 0.001) different from the non–hi-par nodes.

The communities of this network were found after using versatility to choose an optimised resolution parameter (*γ* = 2.0) and are displayed in anatomical coordinates in Fig. 4B. At this resolution parameter, there are 11 modules across the two hemispheres with similar specialisations to those found previously^14^. Rather than select the resolution parameter corresponding to the global minimum of versatility across non-trivial partitions, we selected a value which had consistently low versatility across local perturbations in resolution parameter. The anatomical map of versatility is shown in Fig. 4A. Figure 4D shows the topology of the network, and demonstrates that there are versatile nodes in all communities; versatility is not concentrated in a few communities.

This network was previously found^14^ to have a hierarchical community structure, such that a few large functionally specialised communities were subdivided into smaller sub-communities as the resolution parameter of the Louvain community detection algorithm was incrementally increased. This means that small, fine-scale modules were associated with larger, coarse-grained modules. For example, at fine scales (with higher resolution parameters), the auditory and visual modules were separate, but at higher scales (with a lower resolution parameter) they combined to form the audio-visual module. Most nodes were consistently affiliated to the same community, or one of its offspring sub-communities, over the spectrum of resolution parameters. However, a subset of nodes which also tended to have high participation coefficient (which have previously been coined *hi-par* nodes^14^) were inconsistently affiliated to (sub−) communities in this hierarchy.

Due to the similarity between the criteria for hi-par nodes and the definition of versatility, we hypothesised that the hi-par nodes would have a higher versatility than the non-hi par nodes. As shown in Fig. 4F, versatility is significantly higher for hi-par nodes than it is for non–hi-par nodes; in other words, nodes with high versatility do not fit well into the community hierarchy. This includes nodes in the diencephalon, prefrontal cortex, and basal ganglia; see Supplementary Fig. S5 and Supplementary Table S1 for details.

## Discussion

In a world where community structure is not as black and white as it promises to be, more information than a discrete partition is needed. Community detection algorithms struggle to find a set of non-overlapping communities that adequately represent the community affiliations of the nodes in the network. Furthermore, it is not even clear epistemologically that there *is* a reasonable underlying community structure to any given network; at the extreme end, there is certainly no community structure to an Erdős–Rényi random network, though many algorithms will still yield communities. This is problematic when some of the analyses performed on networks depend highly on the community affiliation of a node, such as the dynamic community structure metrics^24, 25^. This complicates analysis, especially when the change in a node’s community affiliation is the variable of interest.

Rather than seeing this stochasticity as a disadvantage, we used it to define a metric called versatility, which extracts previously discarded information about which nodes are not closely affiliated with any communities. We showed that, while there are many realistic ways to define versatility, our definition (SC) satisfies a number of desirable theoretical properties. In order to ensure compatibility across different situations, fields, and datasets, we explicitly designed versatility to not depend on a specific community detection algorithm, since an algorithm may be chosen for any number of reasons, from computational efficiency to consistency with the literature. Versatility also has connections to fuzzy set theory (see Supplementary Information for more details).

We explored how versatility can be used to choose a resolution parameter for multiscale community detection algorithms based on how much each parameter reduces the ambiguity of the community structure. There is seldom a “correct” resolution parameter because there is seldom a “correct” modular decomposition, and thus there is no possible algorithmic or data driven method which can be used to identify the most meaningful resolution parameter. Instead, versatility can be used to *inform* the selection of a resolution parameter. Newman^26^ recently developed a theoretical formulation of an optimal resolution parameter for *Q*-based algorithms, but the proposed iterative procedure only identifies a single resolution parameter estimate without providing information on its effectiveness or on alternative similarly-effective resolution parameters. We showed that versatility provides a more precise choice in resolution parameter than methods which select a resolution parameter based on the number of communities^12–14^. We also showed that the z-score of the Rand coefficient^10, 15^, while similar to versatility in some cases, cannot be interpreted on the nodal level.

Finally, we examined the versatility of nodes in two networks: the karate club network, and the mouse brain connectome. The karate club network and the mouse brain connectome were both consistent with previous work describing the nodes that do not fit well into any of the modular communities.

Versatility has two core applications for which it is useful. First when used in conjunction with a particular community structure, it is useful as a *post-hoc* method to capture the information about the reliability of community assignment. In this sense, versatility can be seen as a description of the interaction between the algorithm and the network. When using versatility in this way, it is generally desirable to match the algorithm (and the resolution parameter, if applicable) to the network being analysed so that it is possible to determine the reliability with which the information on partition assignment can be interpreted. Second, it is useful in its own right, as a way of finding nodes that do not fit very well with any community. In many cases, these nodes are the most interesting because their interactions are not representative of the community to which they belong, and no community is a good predictor of their interactions. The interpretation of versatility in this case is highly dependent on the particular network.

While the Louvain method for maximising modularity was used extensively in the present work due to its prevalence in the literature, versatility can be applied to any stochastic algorithm which generates a partition across a network. Unlike most community-based methods, versatility is not limited to communities defined by strong intra-modular connections and weak inter-modular connections. For example, recent work^13, 27^ has defined communities based on the similarity between motif profiles. Under these definitions, it is possible for a community to have *no* intra-community edges. In such community definitions, versatility works identically without modification.

Unlike most other community-based methods, versatility does not depend on the naming or identity of particular partitions. Participation coefficient and versatility share superficial similarities but are conceptually very different. Participation coefficient is highly dependent on the particular partition specified by the community detection algorithm, and can often give unintuitive results. This is because participation coefficient relies on the assumptions that “true” underlying communities exist, and that they can be detected by the algorithm. If either of these conditions does not hold, the participation coefficient is difficult to interpret. Participation coefficient was designed for a different purpose—detecting which nodes are important for inter-community communication and linkage—and is able to find inter-community hubs and distinguish them from intra-community hubs^7^. However, it is not successful in uncovering the certainty with which each node can be assigned to a community.

For similar reasons, multi-layer network methods—such as flexibility^24^ and promiscuity^28^—are not well-suited for analysis of single-layer networks. A multi-layer network is an ordered sequence of standard networks (layers) whereby each layer has the same nodes but potentially different edges. A key feature of multi-layer community detection is that community identity is preserved across multiple layers of the network, so that it is possible to (for example) say that node *x* is in community *A* in the first two layers, and then moves to community *B* in the third layer. In order to ensure communities persist across layers, multi-layer community detection algorithms must either optimise the modularity for the entire network at once, treating each node separately and enforcing links between identical nodes in different layers, or run a community detection algorithm on each layer separately and match the communities across layers. In the former case, this enforcement causes less variability in the community assignment between layers. In the latter case, there is no unambiguous way to deal with communities which split into two.

In the words of Kuhn^29^, “The decision to employ a particular piece of apparatus and to use it in a particular way carries an assumption that only certain sorts of circumstances will arise”. Because we have access to algorithms that will separate a network into communities, it is easy to assume that the communities found using these algorithms are “the true underlying communities” and that these communities should be taken as truth in the further analysis of the network. But of course, as we have seen, some assignments may have more truth to them than others.

## Electronic supplementary material

Supplementary Information
