# Supplementary material for: Versatility of nodal affiliation to communities
Source: Sci Rep. 2017 Jun 27;7:4273. doi: 10.1038/s41598-017-03394-5 (PMC5487331; doi:10.1038/s41598-017-03394-5)
Supplement: Supplementary file 1 — Supplementary Information [file 41598_2017_3394_MOESM1_ESM.pdf]

Supplementary information for: Versatility of nodal affiliation to communities

Maxwell Shinn, Rafael Romero-Garcia, Jakob Seidlitz,
František Váša, Petra E. Vértes, Edward Bullmore

Contents

Versatility and fuzzy set theory **1**

Proof of upper bound of `SC` **2**

Proof of lack of upper bound of `EC` **3**

Supplementary figures **4**

Supplementary table **8**

Versatility and fuzzy set theory

Fuzzy set theory[1] is an extension of standard Zermelo-Fraenkel set theory (ZFC) to allow a elements to have a "degree of membership" with a set. In a "crisp" (ZFC) set $A \subset U$ with universal set U, for each element $x \in U$, we must have either $x \in A$ or $x \notin A$. We can define a membership function $\mu : U \to \{0, 1\}$ where $\mu(x) = 1$ iff $x \in A$.

However, in fuzzy set theory, the membership function can have any value between 0 and 1, i.e. $\mu : U \to [0, 1]$. Thus, it is not sufficient to say that x is or is not a member of A; one must specify *to what extent* x is an element of A. Normal set theoretic operations on crisp sets have corresponding fuzzy generalisations. For example, the cardinality of A is $|A| = \sum_{x \in U} \mu(x)$.

Within the framework of fuzzy set theory, the *fuzziness* H of a set, also called *fuzzy entropy* for its similarity to Shannon's entropy[2], quantifies how distant a fuzzy set is from being a crisp set. This is similar in concept to the versatility of a particular node. Thus if we translate the versatility of node j into the notation of fuzzy set theory, we would define A to be the set of all nodes, and its membership function $\mu_j(i)$ to be the probability that node j is in the same community as node i.

Four axioms have become standard criteria to use in defining new measures of fuzziness. Based on the pioneering work of De Luca and Termini[3], the modern axioms are for a fuzziness function H are[1]:

1. For a fuzzy set A with membership function μ, $H(A) = 0$ iff A is crisp, i.e. $\mu(x) \in \{0, 1\}$ for all $x \in A$
2. For a fuzzy set A with membership function μ, $H(A)$ is uniquely maximised if $\mu(x) = .5$ for all $x \in A$
3. For fuzzy sets A and A' defined on the same domain but with different membership functions μ and μ' respectively, if $\mu'(x) \leq \mu(x)$ and $\mu(x) \leq .5$, or if $\mu'(x) \geq \mu(x)$ for $\mu(x) \geq .5$, then $H'(x) \leq H(x)$.
4. Let A^C be defined on the same domain as A with membership function $\mu^{C(x)} = 1 - \mu(x)$. Then $H(A^C) = H(A)$.

Our present definition `SC` satisfies all of these axioms except (4). In the framework for connecting versatility to fuzzy set theory, it is impossible to find a fuzziness measure independent of network size that satisfies axiom (4). The most standard definition of fuzziness, called *normalised fuzzy entropy*, is equivalent to `EU`[3]. Notably, `SU` also satisfies all four of these axioms. (`TU` does not satisfy them because the maximum in (2) is not unique.)

Bridgeness[4] is a network measure related to versatility which is based on the mathematical formalism of fuzzy set theory. However, as it depends on special fuzzy community detection algorithms, it is difficult or impossible to justly compare these metrics to versatility, which operates on traditional community detection algorithms. Thus, this metric was not considered further in the present work.

Proof of upper bound of `SC`

We seek to find an upper bound on versatility (`SC`). To do this, since larger networks allow for higher versatility, we find the limit of the maximum versatility as the network size approaches infinity. In other words, we must solve

$$V_{\max}(j) = \max_{a} \frac{\sum_i \sin(\pi\, \mathbb{E}(a(i,j)))}{\sum_i \mathbb{E}(a(i,j))}. \tag{S1}$$

The maximum of this function will occur when

$$\mathbb{E}(a(i,j)) = \begin{cases} p_0, & i \neq j \\ 1, & i = j \end{cases}$$

for some $0 \leq p_0 \leq 1$. Thus, for a given network size, the maximum versatility for any node in a network of size N is given by

$$V_{\max}(j, N) = \max_{p_0 \in [0,1]} \frac{\sum_{i=1}^{N-1} \sin(\pi p_0)}{1 + \sum_{i=1}^{N-1} p_0}, \tag{S2}$$

and we therefore seek to find

$$V_{\max}(j) = \max_{p_0 \in [0,1]} \lim_{N \to \infty} \frac{\sum_{i=1}^{N-1} \sin(\pi p_0)}{1 + \sum_{i=1}^{N-1} p_0}. \tag{S3}$$

We can see that

$$\begin{aligned}
V_{\max}(j) &= \max_{p_0 \in [0,1]} \lim_{N\to\infty} \frac{(N-1)\sin(\pi p_0) + 0}{(N-1)p_0 + 1} \\
&= \max_{p_0 \in [0,1]} \lim_{N\to\infty} \frac{N\sin(\pi p_0)}{Np_0 + 1} \\
&= \max_{p_0 \in [0,1]} \lim_{N\to\infty} \frac{\sin(\pi p_0)}{p_0 + 1/N} \\
&= \max_{p_0 \in (0,1]} \frac{\sin(\pi p_0)}{p_0} \\
&= \lim_{p\to 0} \frac{\sin(\pi p_0)}{p_0} \\
&= \frac{\pi p_0}{p_0} \\
&= \pi.
\end{aligned}$$

Thus, as the network size approaches infinity, the versatility approaches π.

Proof of lack of upper bound of EC

Using the same construction as in the proof for SC, we want to find

$$V_{\max}(j) = \max_{p_0 \in [0,1]} \lim_{N\to\infty} \frac{\sum_i H(p_0)}{1 + \sum_i p_0}, \tag{S4}$$

where $H(x) = -x\log_2(x) - (1-x)\log_2(1-x)$ for $x \in (0,1)$. We see

$$\begin{aligned}
V_{\max}(j) &= \max_{p_0 \in [0,1]} \lim_{N\to\infty} \frac{NH(p_0)}{Np_0 + 1} \\
&= \max_{p_0 \in [0,1]} \lim_{N\to\infty} \frac{H(\pi p_0)}{p_0 + 1/N} \\
&= \max_{p_0 \in (0,1]} \frac{H(p_0)}{p_0} \\
&= \max_{p_0 \in (0,1]} \frac{-p_0\log_2(p_0)}{p_0} + \frac{-(1-p_0)\log_2(1-p_0)}{p_0} \\
&> \max_{p_0 \in (0,1]} \frac{-p_0\log_2(p_0)}{p_0} \\
&= \max_{p_0 \in (0,1]} -\log_2(p_0).
\end{aligned}$$

But clearly $\max_{p_0 \in (0,1]} -\log_2(p_0) = \infty$. Thus, $V_{\max}(j) = \infty$.

Proofs of the upper bounds of SU, EU, TU, and TC are trivial.

Supplementary figures

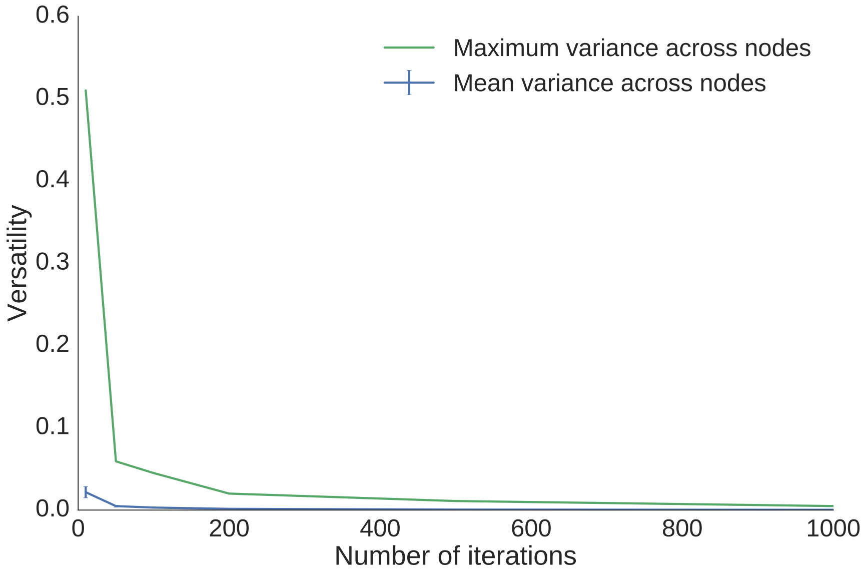

Figure S1: **More iterations of the algorithm increases the stability of versatility**. Versatility was run 10 times at each resolution parameter on the mouse connectome network at a resolution parameter of $\gamma = 2.0$. The variance of versatility for each node was computed. At each resolution parameter, we plot the mean variance in versatility for the average node across networks, as well as the maximum variance in versatility for any node across networks. As the number of iterations increases, the variance decreases. Error bars for the mean variance in versatility are SEM.

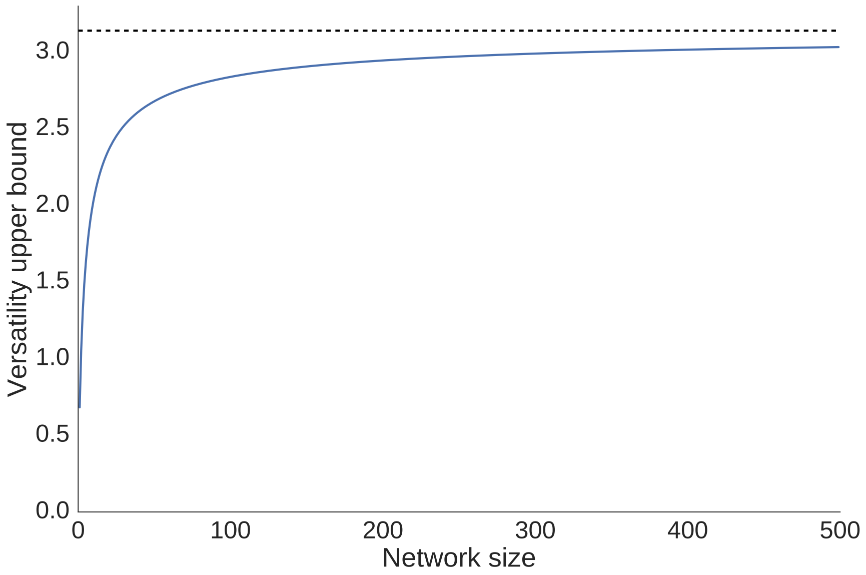

Figure S2: **The upper bound of versatility as a function of network size**. For finite-sized networks, the maximum versatility value of a node is less than π, and is given by the above function. As network size approaches ∞, the maximum versatility approaches π, indicated by the dotted line. This curve was calculated numerically by optimising Equation S2 for each potential network size.

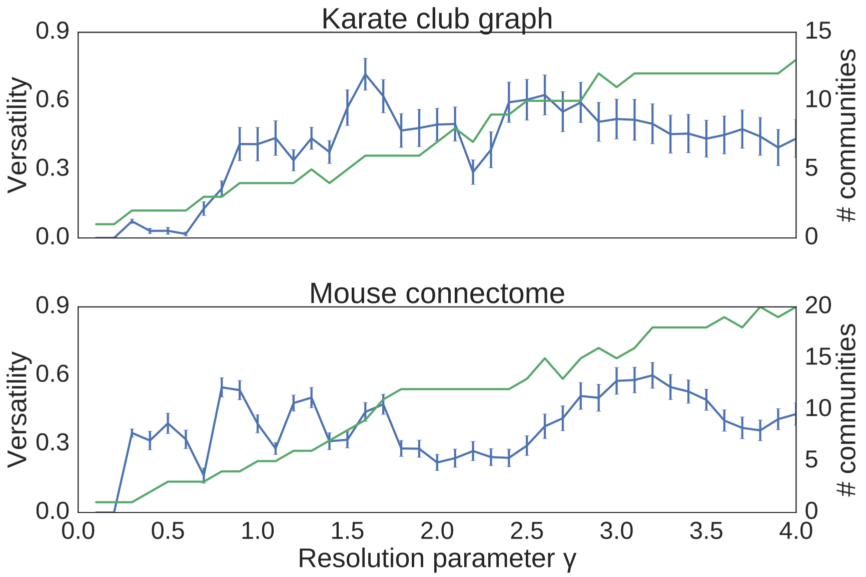

Figure S3: **Versatility compared to number of communities for choosing a resolution parameter.** Recent work[5–7] has used the number of communities as a proxy for choosing a resolution parameter, selecting a resolution parameter from regions where small perturbations in resolution parameter did not change the number of communities detected by the algorithm. This method is compared to versatility above. The blue line shows the mean versatility at each resolution parameter, and the green line shows the number of communities. No unambiguous "plateau" in the number of communities is present in the karate club network at the optimal resolution parameter of 0.5. A "plateau" is present in the mouse connectome network at the optimal resolution parameter of 2.0, but no guidance is provided by this method for choosing a more precise value for the resolution parameter within the 1.8-2.4 range. By contrast, versatility makes precise and meaningful selections in both cases. Error bars on versatility are SEM.

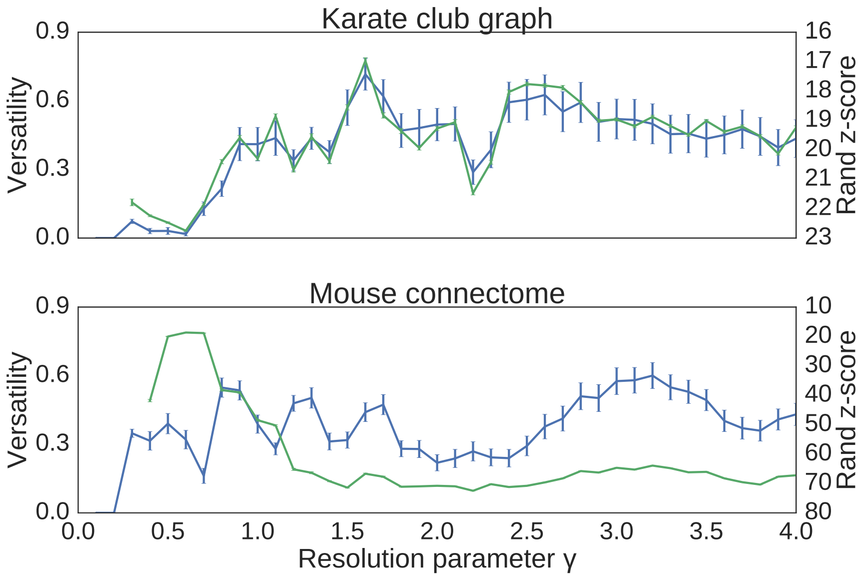

Figure S4: **Versatility compared to the z-score of the Rand coefficient for choosing a resolution parameter.** Recent work[8,9] has used the z-score of the Rand coefficient to guide the choice of a resolution parameter. The blue line shows the mean versatility at each resolution parameter, and the green line shows the Rand z-score. In the Karate Club network, the Rand z-score gives very similar suggestions as versatility. However, there are no obvious local minima in the Rand z-score for the mouse connectome network. Error bars on versatility and Rand z-score are SEM.

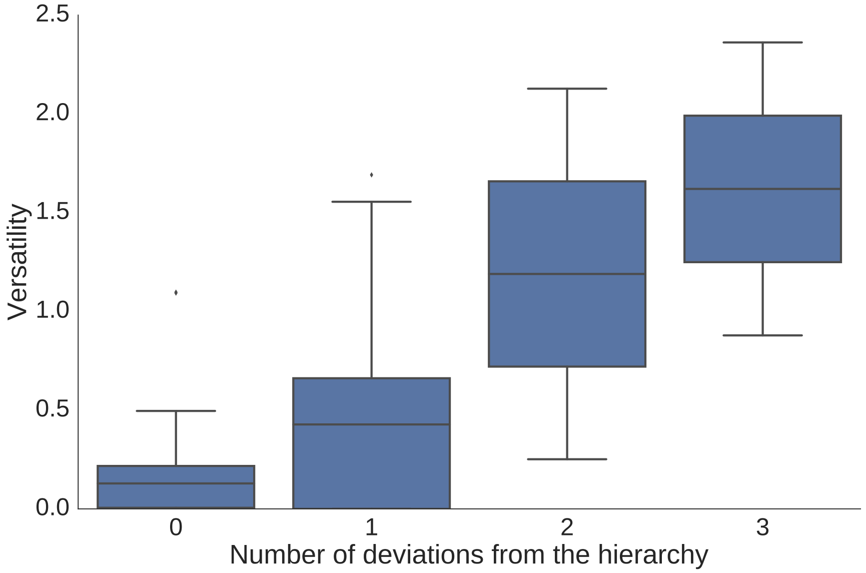

Figure S5: **Nodes with higher versatility have a worse fit into the hierarchy.** In previous work[7], hi-par nodes were defined as those that jumped from one branch of the hierarchy to another on at least one occasion as the resolution parameter was increased. While most of the hi-par nodes in the mouse network only broke from the hierarchy once, two nodes (right and left prosomere 3) broke from the hierarchy twice and two nodes (right and left prosomere 2) broke from the hierarchy three times. We see that these nodes have a higher versatility than those that fit well into the hierarchy, and that hi-par nodes as a whole tend to have a higher versatility than non–hi-par nodes.

Supplementary table

Table S1: **Mouse brain regions and the mean versatility of each region.** For each region, the versatility at $\gamma = 2.0$ was averaged across the two hemispheres. Region names correspond to those described previously[7].

Region name	Versatility
Strp	0.000
ECT	0.000
VISl	0.000
VISpm	0.000
RSP	0.000
VISam	0.000

VISpl	0.000
VISp	0.000
TEa	0.000
VISal	0.000
DG	0.005
CA	0.005
PIR	0.075
CTXsp	0.075
COA	0.075
TR	0.075
AOB	0.075
AON	0.075
RHP	0.075
TT	0.075
AUDv	0.110
PTLp	0.110
AUDp	0.110
AUDd	0.110
GU	0.129
SSp	0.129
SSs	0.129
VISC	0.129
AI	0.131
MOp	0.131
SeSPall	0.216
THy	0.216
PaSe	0.216
POTel	0.216
PHy	0.217
r2	0.217
r1	0.217
CbV	0.217
PMH	0.217
PH	0.217
MH	0.217
is	0.217
CbH	0.219
Dg	0.234
MOs	0.238
ASPall	0.283
ORB	0.427
ACA	0.427
MOB	0.449
ILA	0.482
Stri	0.621
Pal	0.624

m1	0.846
p3	1.189
p1	1.459
p2	1.620

References

[1] Zimmermann, H.-J. *Fuzzy Set Theory-and Its Applications* (Springer Netherlands, Dordrecht, 1996).

[2] Cover, T. M. & Thomas, J. A. *Elements of Information Theory* (Wiley, New York, 1991).

[3] De Luca, A. & Termini, S. A definition of a nonprobabilistic entropy in the setting of fuzzy sets theory. *Information and Control* **20**, 301–312 (1972).

[4] Nepusz, T., Petróczi, A., Négyessy, L. & Bazsó, F. Fuzzy communities and the concept of bridgeness in complex networks. *Phys. Rev. E* **77**, 016107 (2008).

[5] Fenn, D. J. *et al.* Dynamic communities in multichannel data: An application to the foreign exchange market during the 2007–2008 credit crisis. *Chaos: An Interdisciplinary Journal of Nonlinear Science* **19**, 033119 (2009).

[6] Arenas, A., Fernández, A., Fortunato, S. & Gómez, S. Motif-based communities in complex networks. *J. Phys. A: Math. Theor.* **41**, 224001 (2008).

[7] Rubinov, M., Ypma, R. J. F., Watson, C. & Bullmore, E. T. Wiring cost and topological participation of the mouse brain connectome. *PNAS* **112**, 10032–10037 (2015).

[8] Traud, A., Kelsic, E., Mucha, P. & Porter, M. Comparing Community Structure to Characteristics in Online Collegiate Social Networks. *SIAM Rev.* **53**, 526–543 (2011).

[9] Bassett, D. S. *et al.* Robust detection of dynamic community structure in networks. *Chaos: An Interdisciplinary Journal of Nonlinear Science* **23**, 013142 (2013).
